# Analysis of the floral transcriptome of *Tarenaya hassleriana* (Cleomaceae), a member of the sister group to the Brassicaceae: towards understanding the base of morphological diversity in Brassicales

**DOI:** 10.1186/1471-2164-15-140

**Published:** 2014-02-19

**Authors:** Amey Bhide, Simon Schliesky, Marlis Reich, Andreas PM Weber, Annette Becker

**Affiliations:** 1 Justus-Liebig-Universität Gießen, Institute of Botany, Plant Development Group, Heinrich-Buff-Ring 38, 35392 Gießen, Germany; 2 Institute of Plant Biochemistry, Cluster of Excellence on Plant Sciences (CEPLAS) Heinrich-Heine-University, Universitätsstr. 1, D-40225 Düsseldorf, Germany; 3 Department of Biology and Chemistry, University of Bremen, Leobener Str. NW2, D- 28359 Bremen, Germany

**Keywords:** *Tarenaya hassleriana*, *Arabidopsis thaliana*, Floral transcriptome, Cleomaceae, Brassicaceae, Brassicales, 454 sequencing, Anthocyanins, Flower development

## Abstract

**Background:**

*Arabidopsis thaliana*, a member of the Brassicaceae family is the dominant genetic model plant. However, while the flowers within the Brassicaceae members are rather uniform, mainly radially symmetrical, mostly white with fixed organ numbers, species within the Cleomaceae, the sister family to the Brassicaceae show a more variable floral morphology. We were interested in understanding the molecular basis for these morphological differences. To this end, the floral transcriptome of a hybrid *Tarenaya hassleriana*, a Cleomaceae with monosymmetric, bright purple flowers was sequenced, annotated and analyzed in respect to floral regulators.

**Results:**

We obtained a comprehensive floral transcriptome with high depth and coverage close to saturation analyzed using rarefaction analysis a method well known in biodiversity studies. Gene expression was analyzed by calculating reads per kilobase gene model per million reads (RPKM) and for selected genes in silico expression data was corroborated by qRT-PCR analysis. Candidate transcription factors were identified based on differences in expression pattern between *A. thaliana* and *T. hassleriana*, which are likely key regulators of the *T. hassleriana* specific floral characters such as coloration and male sterility in the hybrid plant used. Analysis of lineage specific genes was carried out with members of the fabids and malvids.

**Conclusions:**

The floral transcriptome of *T. hassleriana* provides insights into key pathways involved in the regulation of late anthocyanin biosynthesis, male fertility, flowering time and organ growth regulation which are unique traits compared the model organism *A. thaliana*. Analysis of lineage specific genes carried out with members of the fabids and malvids suggests an extensive gene birth rate in the lineage leading to core Brassicales while only few genes were potentially lost during core Brassicales evolution, which possibly reflects the result of the At-β whole genome duplication. Our analysis should facilitate further analyses into the molecular mechanisms of floral morphogenesis and pigmentation and the mechanisms underlying the rather diverse floral morphologies in the Cleomaceae.

## Background

*Tarenaya hassleriana*, formerly known as *Cleome hassleriana* and sometimes erroneously referred to as *Cleome spinosa*[1] is a quick growing herbaceous perennial, native to Brazil and adjoining South American countries. The species belongs to the section Tarenaya and the subgenus Neocleome within the Cleomaceae [2] which includes roughly 300 species distributed throughout the tropical and subtropical regions of the world [3,4]. The family Cleomaceae belongs to the order Brassicales and previously Cleomaceae were thought to be more closely related to Capparaeceae but recent phylogenetic studies indicate that Cleomaceae are more closely related to and a sister family to Brassicaceae [3,5]. Molecular clock analyses suggests that Cleomaceae and Brassicaceae diverged from each other around 24.2 – 49.4 Million Years Ago (MYA) [6,7].

Analysis of normalized expressed sequence tag (EST) sequences in *T. hassleriana* and comparative genome analysis in *Carica papaya*, both members of the Brassicales, and in *Arabidopsis thaliana* belonging to Brassicaceae revealed that Cleomaceae share the most ancient gamma (γ) whole genome duplication (WGD) with both *C. papaya* and *A. thaliana*. The sister families Brassicaceae and Cleomaceae also share the more recent beta (β) WGD which is lacking in *C. papaya*. However, the third and most recent alpha (α) WGD has occurred independently in Brassicaceae and Cleomaceae. The *T. hassleriana* α WGD (Th- α) is a genome triplication and occurred approximately 13.7 MYA, while the *Arabidopsis thalian*a α WGD (At- α) happened around 23.3 MYA [8]. In spite of the recent Th- α triplication event the genome of *T. hassleriana* is only 1.9 times the size of that of *A. thaliana*[6] and around half the size of the *C. papaya* genome. The small genome size of *T. hassleriana* indicates rapid diploidization, and a faster subsequent gene loss when compared to *A. thaliana*[6].

Cleomaceae are being intensively studied as C4 type photosynthesis evolved de novo in this group of plants. While *A. thaliana* and other Brassicaceae are C3 plants, C4 photosynthesis evolved in Cleomaceae at least three times independently in *Gynandropsis gynandra*, *Cleome oxalidea*, and *Cleome angustifolia. Cleome paradoxa* shows a C3 – C4 intermediate anatomy and physiology thus making Cleomaceae a model system to study C3 – C4 evolution [9]. Comparative leaf transcriptome studies by RNA-Seq have been carried out in *G. gynandra* (C4) and *T. hassleriana* (C3) to elucidate and identify novel genes and gene networks responsible for the C4 anatomy [10].

**Figure 1:**
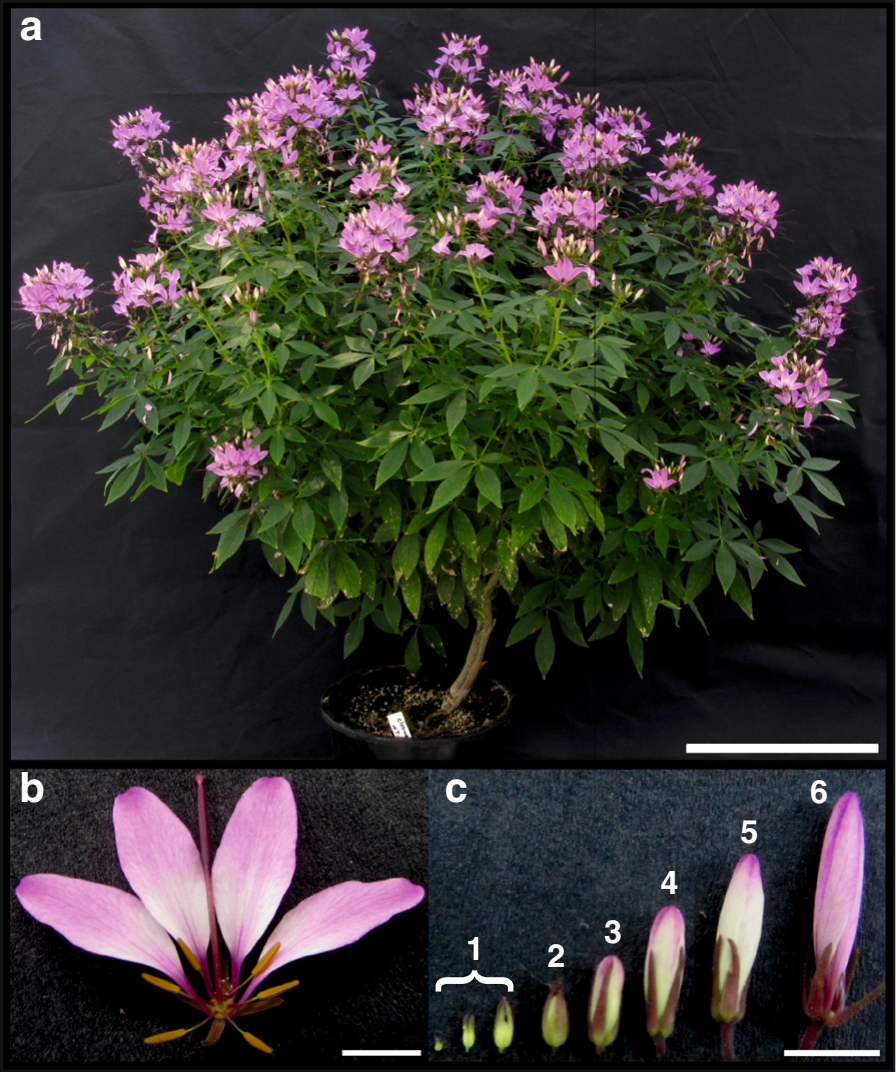
***Tarenaya hassleriana *plant. a)** Morphology of a flowering *T. hassleriana* plant. **b)** Flower at anthesis showing four small purple sepals, four showy pink petals, six yellow stamens and a central gynoecium. **c)** Bud stages 1–6 characterized by bud length, bud stage 1 (<2.5 mm), bud stage 2 (3–4 mm), bud stage 3 (4–5 mm), bud stage 4 (6–8 mm), bud stage 5 (8–10 mm), bud stage 6 (12–15 mm). Scale bar 30 cm for a and 5 mm for b and c.

*T. hassleriana* (Figure 1) is also called the spider flower plant due to the long stamens which appear like appendages of spiders and is a popular ornamental plant owing to its colorful and abundant flowers. Adult plants can grow about five feet tall and several feet in diameter with several lateral branches. The stem and the lateral branches are soft and succulent but the main stem and older branches become woody with age. The leaves are palmate with 3–5 folioles per leaf (Figure 1a). Plants start flowering while they are in the juvenile stages and most of vegetative growth overlaps with the flowering period [11].

A typical *T. hassleriana* flower is zygomorphic unlike the disymmetric *A. thaliana* flower. Each flower has four sepals, four petals, six stamens, and a single gynoecium composed of two fused carpels (Figure 1b). The flower buds are laid out in a disymmetric bauplan during the early developmental stages which changes near anthesis and the mature flowers become zygomorphic. Conversely, in *A. thaliana* early developmental bud stages are monosymmetric and the flowers become disymmetric near anthesis [12]. *C. papaya* flowers on the other hand are actinomorphic at anthesis but early development has not been characterized yet. *T. hassleriana* inflorescences produce hermaphroditic, female, or male only flowers such that fruits are only periodically formed. The synchronous and alternate appearance of male, female, and hermaphroditic flowers in a raceme favors out-crossing, and prevents selfing except in the case of the hermaphroditic flowers [11]. This feature distinguishes *T. hassleriana* from most plants which are either dioecious (like *C. papaya*) with separate male and female plants which rarely produce hermaphroditic flowers, or monoecious like *A. thaliana* which is an obligate self-pollinated plant with hermaphroditic flowers. *T. hassleriana* plants are very prolific, they reseed and establish in suitable environments very easily and escape from cultivation, often becoming invasive in subtropical countries like Japan, New Zealand, parts of Australia, and the United States of America [13]. Hence many horticultural varieties, possibly like the one used in this study were developed to be sterile so that they cannot establish in non-native environments.

Also unlike most Brassicales *T. hassleriana* flowers are very colorful due to the presence of various anthocyanins and show ‘petal fading’ i.e. loss of pigmentation and dry matter associated with anthesis. This phenomenon coupled with favored cross pollination may suggest a specific role in flower - pollinator interactions or simply an age related phenomenon [14].

A close relationship to *A. thaliana* facilitates the analysis of *T. hassleriana* specific traits, such as flower coloration, alternating development of three types of flowers and flexible shifts from vegetative to reproductive growth, which are all not found in *A. thaliana*. Here, we describe the floral transcriptome sequence along with Transcriptome Sequencing Expression (TSE) of a horticultural *T. hassleriana* hybrid as a starting point for further analysis of Tarenaya flower development. Expression analysis by qRT-PCR documents the robustness of the TSE and rarefaction analysis shows that the transcriptome sequencing covers even rare transcripts. Candidate genes that may be involved in the *T. hassleriana*-specific flower developmental processes have been identified and are presented here.

## Methods

### Plant material and growth parameters

A *T. hassleriana* hybrid plant was obtained from a local garden center. It was grown in 3:1 mixture of a peat and sand based potting soil with perlite supplemented with 2 g/l Osmocote® slow release fertilizer (Scotts Deutschland GmbH, Nordhorn, Germany). The plant was grown in a greenhouse under long day growth conditions (17 hours light and 7 hours dark) with light varying between 80 and 700 μmol.m^-2^.s^-1^ photons. Supplemental lighting of 70 μmol.m^-2^.s^-1^ was provided throughout the photoperiod. The temperatures in the green house varied between 20°C (day) and 16°C (night).

### Tissue collection, nucleic acid extraction, and cDNA synthesis

*T. hassleriana* floral tissue was collected for RNA and DNA extraction. The collected flower tissue was composed of equal quantity (by mass) of flowers at anthesis and each of the 6 floral bud stages defined by bud length as shown in Figure 1c).

For the floral transcriptome sequencing total RNA was extracted from the *T. hassleriana* floral tissue using guanidium thiocyanate-phenol-chloroform extraction protocol [15]. The polyA^+^ mRNA was isolated using the Oligotex mRNA Minikit (Qiagen, Hilden, Germany) according to the manufacturer’s instruction. The purified mRNA was analyzed for quality and quantity using the Eukaryote Total RNA Pico assay of the Agilent 2100 Bioanalyzer (Agilent Technologies, Böblingen, Germany). For the qRT-PCRs total RNA was isolated from floral tissues with the plantRNA Kit-OLS® (Omni Life Science, Bremen, Germany) following the manufacturer’s protocol. Genomic DNA was extracted from the floral tissue using the DNeasy® Plant Mini kit (Qiagen, Hilden, Germany) according to the manual.

### Library preparation and 454 pyrosequencing

200 ng of purified polyA^+^ mRNA was used to synthesize the cDNA for sequencing with Roche Rapid Library kit (Roche) following the manual. A massively parallel pyrosequencing run was performed on a GS FLX using Titanium chemicals (Roche) with a split picotiterplate allowing two replicates to run at the same time.

### Assembly, annotation and gene expression

All reads together were de novo assembled using CLC Genomics Workbench 4.9 (clcBio, Aarhus Denmark). Default parameters were chosen for the assembly. The resulting 49321 contigs were annotated against TAIR10 coding sequences (representative gene model 20110103). A reciprocal BLATX mapping was performed [16] and the best bi-directional hit per contig was kept as annotation. Chimeric contigs were determined with the pipeline provided in [17].

Gene expression was determined by mapping the reads to TAIR10 coding sequences using BLATx. The single best hit for each read was counted. Expression values were normalized to reads per Kilobase gene model per mappable million (RPKM). All reads were additionally mapped to the *T. hassleriana* floral transcriptome contigs with CLC Genomics Workbench for subsequent rarefaction analysis. The expression data for the *T. hassleriana* leaf transcriptome was obtained from Bräutigam et al. [10].

### Lineage specific gene detection

Based on the *T. hassleriana* floral contigs, mappings to the transcriptomes of *A. thaliana*, *B. rapa*, *C. papaya*, and *P. trichocarpa* with an e-value cutoff of 10^-10^ were created in proteinspace. From those all against all mappings the 15 overlapping sets and the residual *T. hassleriana* specific set were determined using R’s set methods [18].

### Quantitative reverse transcription PCR (qRT-PCR)

For the qRT-PCRs the first strand cDNA was synthesized with the RevertAid™ H Minus First Strand cDNA Synthesis Kit (Fermentas, St.Leon-Rot, Germany) according to the manufacturer’s protocol using an universal oligo(dT) (T_18_) primer. qRT-PCR experiments were performed according to the MIQE guidelines [19]. Exon spanning primers were then generated using PerlPrimer 1.1.21. [20]. A primer efficiency test was carried out and all the primers were tested with genomic DNA to ensure cDNA specificity. (Primer sequences are provided in Additional file 1: Table S2).

The qRT-PCR assay was performed in 96 well plates using the LightCyler®480 II (Roche, Mannheim, Germany) and analyzed with the LCS480 1.5.0.39 software. Each reaction was composed of 10 μl of 2x DyNAmo™ Flash SYBR® Green qPCR Mastermix (Biozym Scientific GmbH, Oldendorf Germany), 2 μl each of 10 μM forward and reverse primers, 1 μl H_2_O and 5 μl of diluted cDNA template. Standard dose response (SDR) curves were constructed for all the genes by using serial dilutions (1:50 to 1:50,000) of the cDNA template. Each reaction was performed in biological duplicates and technical triplicates along with water and RNA controls for each primer pair. The *T. hasslerianaACTIN7 (ACT7)* gene served as an internal control. The following PCR program was used: 7 min at 95°C; 45 cycles of 10 s at 95°C, 15 s at 60°C, 15 s at 72°C, followed by a melting curve of 5 s at 95°C, 1 min at 65°C and 30 s at 97°C. The Absolute Quantification analysis and the quantification cycle (Cq) were calculated according to the Fit Points method using the LCS480 1.5.0.39 software. The amplification efficiency was calculated using the SDR for each gene. The raw data were analyzed according to the relative standard curve method and the fold difference between the expression of *ACT7* and the genes of interest was calculated using the comparative Cq method (ΔΔCq) [21]. A one way ANOVA was performed to calculate the statistical significance of the difference between the three expression values.

### Comparison of *A. thaliana* and *T. hassleriana* floral gene expression and GO annotations

In order to identify genes that may play a role in the *T. hassleriana* specific floral traits, transcripts specific for the *T. hassleriana* flower, not expressed in the *A. thaliana* flower and vice versa were identified. Microarray expression data [22] for *A. thaliana* flower stages 1–6, 9, 10–11, 12, 15 (ATGE_29_A2, B2, C2; ATGE_31_A2, B2, C2; ATGE_32_A2, B2, C2; ATGE_33_A2, B2, C2; ATGE_39_A2, B2, C2) were downloaded from

The Arabidopsis Information Resource (TAIR), http://arabidopsis.org/servlets/TairObject?type=hyb_descr_collection&id=1006710873#497, on, Nov 20 2012.

Of the 22,746 microarray probes hybridizing to 23,570 genes, only 21,107 probes hybridizing to unique transcripts were considered for the analysis. A dataset corresponding to the expression of these 21,107 transcripts in the *A. thaliana* floral transcriptome was compiled. Expression of a gene in at least one floral stage and sample subset was considered as presence of the transcript in the *A. thaliana* floral transcriptome. The presence or absence of homologous transcripts in the *T. hassleriana* floral transcriptome was analyzed. A list of putative *T. hassleriana* orthologs of *A. thaliana* genes expressed in *T. hassleriana* floral transcriptome but not in the *A. thaliana* floral transcriptome was constructed. Also, transcripts present in the *A. thaliana* flower transcriptome but homologs absent in the *T. hassleriana* transcriptome were identified. Gene Ontology (GO) annotations were assigned to genes expressed exclusively in the *A. thaliana* or *T. hassleriana* transcriptome using the online tool for functional annotation Blast2GO® [23] by performing a BLASTX with a cutoff value of 1e^-100^ as this value showed robust matches of GO annotations to TAIR annotations.

GO annotations were assigned to *T. hassleriana* lineage specific sequences, and other sequences shared by Cleomaceae with the Brassicaceae, Brassicales or lost in the Brassicaceae using Blast2GO® [23] by performing BLASTX and BLASTN with cutoff values of 1e^-10^.

### Rarefaction analysis

Rarefaction analysis is commonly used in ecological research defining species richness as a function of sequencing effort. Such an analysis can be broadened to genomics as long as the data are distributed as described in the original paper defining the underlying equation [24]. Hale et al. [25] already calculated rarefaction curves for transcriptome analysis of a polyploid lake sturgeon. Here, we applied rarefaction analysis to ascertain whether sequencing depth and coverage was sufficient to draw a comprehensive picture of the transcriptome of Cleome. Thus, three different libraries were created: one data set for each biological replica as well as a merged one. Data sets were constructed by listing each gene (defined by a contig) with its read support. Rarefaction curves were calculated using the program aRarefactWin (https://www.uga.edu/strata/software/). Hereby, genes were randomly resampled and it was recorded which gene of the library was identified with which frequency. This procedure was repeated 1,000 times. Then, the average number of each gene found was plotted for different read numbers drawing a curve whose slope indicated if sequencing effort was deep enough. This was the case when the curve flattened and ran into a plateau.

## Results

### Sequencing

Massively parallel pyrosequencing of two samples of *T. hassleriana* (Additional file 1: Table S1) yielded 1,254,286 sequencing reads in total. The sequencing raw data are deposited in the DDBJ (DNA Data Bank Japan, http://trace.ddbj.nig.ac.jp/index_e.html) under the experiments SRR1051360 and SRX393170 https://trace.ddbj.nig.ac.jp/DRASearch/run?acc=SRR1051360 and (https://trace.ddbj.nig.ac.jp/DRASearch/experiment?acc=SRX393170) The histogram of reads by length (Additional file 2: Figure S1a) showed an average read length of ~316 nucleotides. Roughly 45% of the reads could be mapped against *A. thaliana* TAIR10 coding sequences for counting gene expression.

Assembling the reads de novo resulted in 49,237 contigs with an N50 of 690 bases (Additional file 2: Figure S1b). Of these, 41,320 could be annotated by mapping against Arabidopsis. 1.1% (537) chimeric contigs could be detected in the assembly.

### Rarefaction analysis

**Figure 2:**
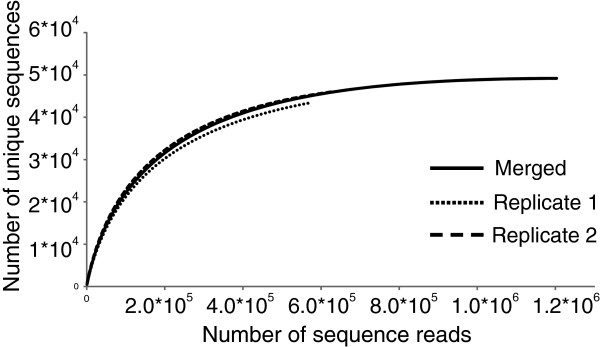
**Rarefaction for transcriptome depth and coverage analysis.** Rarefaction curves for both libraries representing expected gene coverage rates. Curves were constructed for the two biological replicates 1 and 2 and a merged sample.

Rarefied libraries were constructed separately for the two biological replicates 1 and 2 and a merged sample to illustrate possible differences in gene discovery rates. Although the gene discovery rate of replicate 1 was less than the one of replicate 2 (Figure 2), the curves for both replicates indicated that a larger part of the *T. hassleriana* floral transcriptome was detected as the curves already flattened. However, the merging of the information of both libraries affected the overall output as the rarefaction curve reached nearly a plateau (Figure 2). This shows that each library comprised genes not detected with the other one. Thus, the merged data set allows drawing a detailed view of the transcriptome of *T. hassleriana*. Increasing the sequencing depth would only result in the detection of extremely rare genes.

### qRT-PCR expression analysis validates transcriptome sequencing expression (TSE)

**Figure 3:**
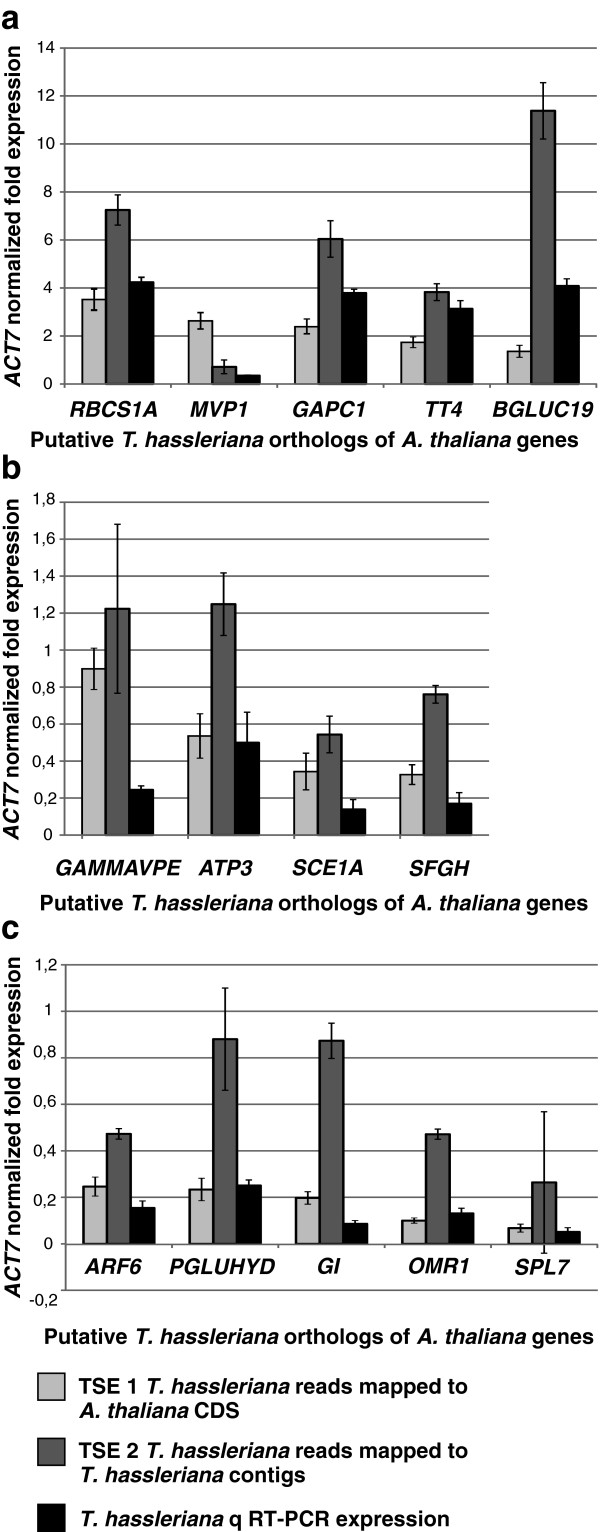
**Comparative analysis of Transcriptome Sequencing based Expression data (TSE) with qRT-PCR expression data.** The TSE and the qRT-PCR expression were normalized with the putative *T. hassleriana ACTIN7* homolog. The first column represents the TSE 1 where the *T. hassleriana* reads are mapped on to the *A. thaliana* CDS sequences; the second column represents the TSE 2 where the *T. hassleriana* reads are mapped on the *T. hassleriana* contigs and the third column represents the qRT-PCR expression data. **a**: Comparison of TSE and qRT-PCR in genes with high expression (750–2000 RPKM, **b**: Comparison of TSE and qRT-PCR in genes with moderate expression (150–300 RPKM) and **c**: Comparison of TSE and qRT-PCR in genes with low gene expression (25–150 RPKM). The error bars represent the standard deviation and the P-values for statistical significance between expression values are presented in Additional file 1: Table S4.

**Figure 4:**
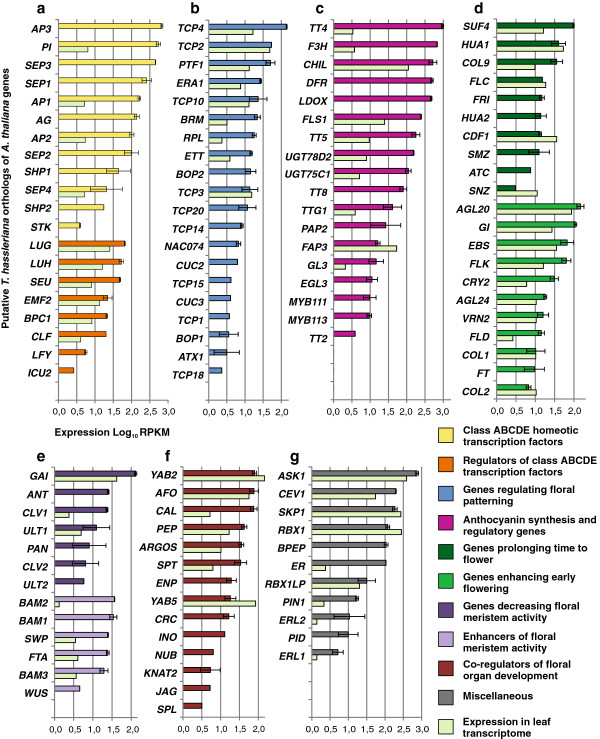
**Expression of *T. hassleriana *orthologs of *A. thaliana *genes regulating various floral characteristics in the *T. hassleriana *flower and leaf transcriptomes.** The putative orthologs are plotted on the Y axis and the Transcriptome Sequencing Expression (TSE 1) which is the log_(10)_ of RPKM is plotted on the X axis. The error bars show standard deviation; **a**: Expression of class ABCDE homeotic transcription factors and their regulators in the floral and leaf transcriptomes of *T. hassleriana*, **b**: Genes regulating patterning and symmetry, **c**: Genes involved in synthesis and regulation of anthocyanins, **d**: Genes regulating time to flower, **e**: Positive and negative regulators of floral meristem activity, **f**: Co-regulators of floral organ development, **g**: Miscellaneous group genes involved in flower development.

The robustness of expression data generated by the transcriptome sequencing was analyzed independently using a qRT-PCR assay (Figure 3). A normalized expression profile for *T. hassleriana* reads mapped to *A. thaliana* CDS sequences was created by calculating the ratio of reads mapped to an individual gene against the reads mapped to *A. thaliana ACT7*. A subset of 14 genes was randomly chosen to represent genes with high (normalized expression ratio 1.0 – 10.0, Figure 4a), moderate (normalized expression ratio 0.3 – 1.0, Figure 4b) and low (normalized expression ratio 0.05 – 0.3, Figure 4c) expression levels. The expression of the putative *T. hassleriana* orthologs of the *A. thaliana genes RBCS1A, MVP1, GAPC1, TT4, BGLUC19, GAMMAVPE, ATP3, SCE1A, SFGH, ARF6, PGLUHYD, GI, OMR1*, and *SPL7* was analyzed in *T. hassleriana* floral tissue (*A. thaliana* gene identifier, full gene names are shown in Additional file 1: Table S3). The qRT-PCR expression data were also normalized to the expression of the *T. hassleriana ACT7*.

Generally we found a better match of transcript abundance detected by qRT-PCR in *T. hassleriana* as compared to reads mapped to the *A. thaliana* orthologs (TSE1) than to the *T. hassleriana* contigs (TSE2). A correlation plot for the comparison of expression measured by qRT-PCR and TSE was generated (Additional file 2: Figure S3). When all the 14 gene expressions by the two methods were plotted a positive linear correlation was observed (Additional file 2: Figure S3a) as indicated by a R^2^ value 0.55. The expression of *MVP1* and *BGLUC19* gene homologs which belong to big gene families with 41 and 66 homologs in *A. thaliana* respectively was the most significant outlier in this plot. When the expression data for the *MVP1* and *BGLUC19* gene homologs were removed and the data plotted again a very strong positive linear correlation between TSE1 and qRT-PCR expression values was obtained with an R^2^ value 0.91 (Additional file 2: Figure S3b). This indicated that TSE1 approach for measuring gene expression was very robust except for genes belonging to large gene families with highly similar homologs in which case the read mapping may be incorrect. Nonetheless a positive linear expression correlation for all genes corroborates the TSE1 expression data. In particular, similar normalized fold expression between qRT-PCR data and reads mapped to the *A. thaliana* orthologs were observed in the genes *RBCS1A* (high expression), *ATP3, SCE1A, SFGH* (moderate expression), and *ARF6, PGLUHYD, OMR1*, and *SPL7* (low expression) P >0,01 (Additional file 1: Table S4 shows the comparative P values for the ANOVA tests). In case of *T. hassleriana* homologs of genes *GAMMAVPE, MVP1* and *TT4* the transcript abundance detected by qRT-PCR was more similar when reads were mapped to the *T. hassleriana* contigs (TSE2) P > 0,01. In case of the *GAPC1* and *BGLUC19* homologs the difference between qRT-PCR expression and TSE1 and TSE2 was statistically significant P < 0,01. It was further observed that the number of reads mapped to the *T. hassleriana* contigs was in all cases, with the exception of *TT4*, grossly overestimating gene expression.

### Expression of genes controlling floral traits in the flower and leaf transcriptome

Genes controlling various floral traits and flower development in *A. thaliana*, *Antirrhinum majus*, *Fagopyrum esculentum* etc. were identified based on literature [26-31]. The expression pattern of their putative *T. hassleriana* orthologs identified by a bidirectional BLATX search with the *A. thaliana* CDS sequences was analyzed in the flower and leaf transcriptomes to learn more about the regulation of the special floral traits of *T. hassleriana* (Figure 4). The selected genes were first grouped into different classes such as homeotic transcription factors, regulators of homeotic genes etc. and ordered within their groups according to transcript abundance. Of the genes analyzed, 49 (41.9%) were specific to the flower transcriptome and not found in the leaf transcriptome. (*A. thaliana* gene identifier, full gene names are shown in Additional file 1: Table S3).

Amongst the putative class ABCDE homeotic transcription factor orthologs, the highest expression was observed among the class B gene homologs *AP3* and *PI* and the class E gene homologs *SEP1* and *SEP3*. The putative ortholog of the C class gene *AG* was expressed at a 10 fold lower magnitude compared to the class B and E genes. The expression of the putative orthologs of the D class genes *SHP1*, *SHP2* and *STK* the expression of which regulates the ovule and fruit development in *A. thaliana* was found to be considerably lower, when compared to the class ABCE genes. *AP3, SEP3, SEP1*, and *STK* transcripts were not present in the leaf transcriptome while *PI, AP1, AP2*, and *SEP4* are expressed at a very low level in leaves. In addition to these, 25 other putative MADS box transcription factors without floral homeotic function that are members of the MIKC, Mα, Mβ, Mγ, Mδ subfamilies were also found to be expressed in the floral transcriptome.

Amongst the genes putatively regulating the class ABCDE homeotic transcription factors, the *LUG, LUH* and *SEU* orthologs showed highest expression in the flower transcriptome, while a 10 fold lower expression of these genes was observed in the leaf transcriptome. The expression of the *LFY* homolog was also observed in the floral transcriptome albeit at very low levels. Interestingly, putative homologs of genes regulating class B gene activities like *UFO* and *SUP* and class A gene activity like *SAP* were not identified in the floral transcriptome library. The orthologs for genes regulating patterning and symmetry also showed expression in the floral transcriptome. The putative orthologs of *TCP4, TCP2*, and *PTF1* showed the highest expression. The expression of these genes was also observed in the leaf transcriptome; in case of the *TCP4* ortholog a 100 fold higher expression was observed in the floral transcriptome when compared to the leaf transcriptome, while the *TCP2* ortholog expression was almost equal in both the transcriptomes. Comparatively low expression of other putative patterning gene orthologs like *TCP14, TCP15, TCP18, CUC2* and *CUC3* was also observed specific to the floral transcriptome.

While the *A. thaliana* flowers are mostly free of pigments, the petals and reproductive organs of *T. hassleriana* are pink and dark magenta and hence the expression of putative orthologs of genes regulating anthocyanin production, regulation, and deposition was analyzed. Very high expression was observed for the putative orthologs of *TT4, F3H, CHIL, DFR* and *LDOX*. Most genes show a higher expression in flowers than in leaves and for several, such as *DFR, LDOX*, and *TT8*, expression is specific to the flower suggesting key roles in flower pigmentation. Very low expression of *TT4* and *F3H* orthologs (about 300 and 200 fold lower respectively) was observed also in the leaf transcriptome, whereas the *CHIL* ortholog expression was only about 5 fold lower in the leaf transcriptome. The spatiotemporal expression pattern of *A. thaliana* orthologs of these genes was investigated in *A. thaliana* using the Arabidopsis eFP Browser (http://bar.utoronto.ca/efp/cgi-bin/efpWeb.cgi) [32]. The expression patterns for the homologs of *TT4, F3H, CHIL*, and *FLS1* was very similar in *T. hassleriana* and *A. thaliana*. The enzymes encoded by these genes are required for the synthesis of flavonoids like quercitin, dihydroquercitin, myricetin etc., which are intermediates of anthocyanin biosynthesis. The products of the genes *DFR, LDOX, UGTD2* which were found to be expressed in in the *T. hassleriana* floral transcriptome but only in senescing leaves in *A. thaliana* (Table 1) are involved in downstream processes that catalyze the conversion of the flavonoids into anthocyanins like Pelargonidin and Cyanidin which determine the characteristic pink-magenta flower color. Genes like *PAP2*, *MYB111*, *MYB113*, and *EGL3* are regulators of flavonoid and anthocyanin biosynthesis and were also expressed in *T. hassleriana* floral tissue whereas in *A. thaliana* their expression was restricted to senescing leaves and seeds during early stages of embryo development.

**Table 1:** **Genes putatively involved in anthocyanin synthesis, regulation, and deposition found in the floral transcriptome of *T. hassleriana *** **and the expression of their putative orthologs in ***A. thaliana*** tissues and developmental stages**

**Gene homologs expressed in *T. hassleriana *** **floral transcriptome**	**Expression in *A. thaliana***
*TT4*	Buds, senescent leaf, seed (globular embryo stage)
*F3H*	Buds, petal, seed (globular and torpedo stage embryo)
*CHIL*	Buds, petal, young silique, seeds (globular and torpedo stage embryo)
*DFR*	Senescent leaf, young silique, seed (heart stage embryo)
*LDOX*	Senescent leaf, young silique, seed (heart stage embryo)
*FLS1*	Buds, petal, seeds (torpedo and walking stick stage embryo)
*TT5*	Buds, petal, carpel, seed (globular and heart stage embryo)
*UGTD2*	Senescent leaf, seed (curled cotyledon, green cotyledon stage embryo)
*UGTC1*	Senescent leaf
*TT8*	Young siliques, seeds (heart, walking stick stage embryo)
*TTG1*	All plant organs, high expression in cauline and senescent leaves, young siliques, seeds (heart and torpedo stage embryo)
*PAP2*	Senescent leaf
*FAP3*	Cauline leaf, young siliques, seeds (Heart, torpedo, walking stage embryo)
*GL3*	Expression data not available
*EGL3*	Shoot apex (vegetative, floral transition, inflorescence), young silique, seeds (globular, torpedo, walking stick stage embryo)
*MYB111*	Petals, shoot apex (inflorescence)
*MYB113*	All plant organs, high expression in pollen, seeds (curled cotyledon and green cotyledon embryo stage)
*TT2*	Young siliques, seeds (globular and heart stage embryo)

Expression of gene orthologs governing time to flower was also analyzed. Expression of both antagonistic groups of genes that prolong time to flower or enhance the transition into flowering was observed. Among the orthologs inducing flowering *AGL20, GI, EBS*, and *FLK* had the highest expression; expression of these genes was also observed in the leaf transcriptome at very comparable levels. Amongst the orthologs of genes delaying flowering *SUF4, HUA1, COL9*, and *FLC* had high levels of expression which was also observed at comparable levels in the leaf transcriptome. The orthologs of *FRI, HUA2, SMZ*, and *ATC* showed moderate to low floral transcriptome specific expression.

*T. hassleriana* homologs of meristem activity regulators, such as *GAI, ANT* and *CLV1* which are involved in decreasing meristem proliferation was observed at high levels in the flower and varying levels in the leaf transcriptome while *ANT* expression was not detected in the leaf transcriptome. Putative homologs of genes *BAM1, BAM2, BAM3* and *WUS* which enhance meristem proliferation were also found to have moderate expression levels in the floral transcriptome. Interestingly, putative homologs for *FTA*, *ERA1*, and *STM*, were found to be expressed in the floral transcriptome as their *A. thaliana* counterparts show very low expression the flower.

Another important category of gene orthologs analyzed for expression are the genes that co-regulate floral organ development alongside the ABCDE floral homeotic transcription factors. High expression was observed in case of orthologs of *YAB2, AFO* and *PEP* in both the floral and leaf transcriptomes whereas the expression of the *CAL* ortholog was about 100 fold higher in the flower transcriptome. Other floral organ developmental regulators, such as *ENP, CRC, INO, NUB, JAG*, and *SPL* were not identified in the *T. hassleriana* leaf transcriptome, but only in floral transcriptome whereas they are also expressed in *A. thaliana* leaves at very low levels. No expression was observed for *ROXY* gene homologs which are responsible for anther and male gametophyte development downstream of *SPL*.

Other putative homologs of *A. thaliana* floral regulators were identified amongst them were the highly expressed homologs of genes *ASK1, CEV1, SKP1, RBX1*, which are part of SCF ubiquitin protein ligase complexes which regulate multiple aspects of flower development together with *UFO* in *A. thaliana*[33]. Homologs of genes *ER, ERL1* and *ERL2* which are protein kinases that influence meristem cell fate and patterning in the inflorescence meristem were also highly expressed. Interestingly the homolog of *BPEP* was found to be expressed only in the floral transcriptome, while the two distinct *BPEP* transcripts in *A. thaliana* are expressed in the floral as well as in vegetative organs respectively. Homologs of genes *PIN1* and *PID* were also expressed which are known to affect size, floral organ number and total number of flowers in *A. thaliana*.

This in silico expression analysis of genes related to flower development demonstrates that with the chosen RNAseq method we are able to monitor gene expression in logarithmic scales covering more than two magnitudes. In addition, the two library preparations for this sequencing experiment show only rarely any difference in RPKM. Detailed expression analysis of putative *T. hassleriana* homologs of *A. thaliana* genes in the *T. hassleriana* floral transcriptome is provided in Additional file 3 along with the AGI identifiers.

### Characterization of genes putatively governing sterility in *T. hassleriana*

**Figure 5:**
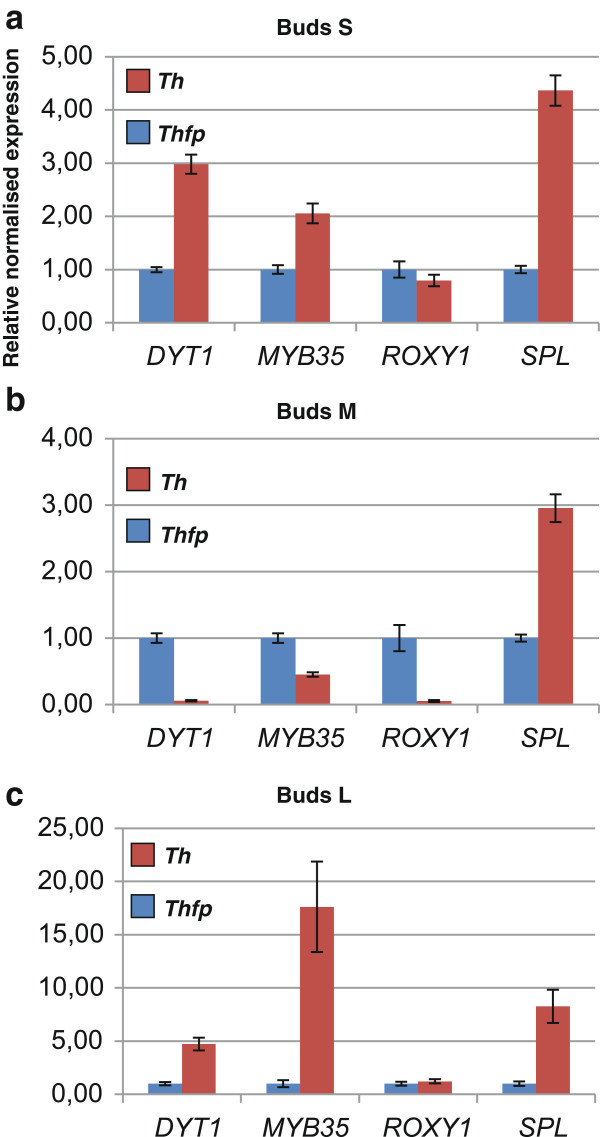
**Comparative expression analysis of genes governing stamen development and microsporogenesis in the sterile *T. hassleriana hybrid *** **(Th) and the fertile *T. hassleriana *** **parent (Thfp) in a: Small buds, stage 1 (S), b: Medium sized buds, stage 2 and 3 (M), and c: Large buds (stage 4 to 6) and open flowers, (L).**

The particular *T. hassleriana* hybrid used in this study was sterile. While orthologs of *A. thaliana* regulators of anther development were expressed in the *T. hassleriana* flower, no expression of *ROXY1* and *ROXY2* was detected. These two genes redundantly control the anther lobe and pollen mother cell differentiation downstream of *SPL*[34]. The genome of one of the parents of this hybrid, *T. hassleriana* Purple Queen (ES1100) was recently published [35] and this plant, unlike its hybrid offspring is fertile. Only *ROXY1* ortholog was found in the *T. hassleriana* genome To learn more about the possible causes for the sterility we compared the expression pattern of homologs of *SPL, ROXY1* and their *A. thaliana* downstream targets *DYT1* and *MYB35* affecting stamen development and microsporogenesis in these two plants by qRT-PCR at small, medium and large buds (Figure 5).

Expression analysis by qRT-PCR indeed revealed that the expression of the *ROXY1* homolog was very low (10^3^ fold lower compared to *ACT7*) and well beyond the scope of detection by RNA seq. *ROXY1* expression was down regulated in the sterile hybrid only at bud stage M when compared to the fertile parent the (Figure 5b) whereas it was similar to the parent at the younger and later developmental stages. Along with the down regulation of *ROXY1*, expression for the *DYT1* and *MYB35* homologs which most likely act downstream of *ROXY1* was also down regulated in stage M buds. In stages other than M, the expression of *DYT1* and *MYB35* homologs in the sterile *T. hassleriana* hybrid was several fold higher than the respective expression in fertile parent buds in both the early and late developmental stages. Expression of the *SPL* homolog in the sterile hybrid buds was 3–4 fold higher than the fertile plant buds in stages S and M whereas in stage latter L the expression was 8 fold. Thus our expression data suggest that the complex network governing stamen development and microsporogenesis is disrupted in the *T. hassleriana* hybrid which could provide a causal link to its sterility.

### Characterization of *T. hassleriana* floral transcriptome specific genes in comparison to *A. thaliana*

We described above that the flower of *T. hassleriana* is morphologically distinct from the *A. thaliana* flower and our aim was to identify genes that may contribute to the differences by comparing the *A. thaliana* floral transcriptome with that of *T. hassleriana*. However, as our data are based on RPKM and the *A. thaliana* are microarray data the two datasets may be compared only qualitatively but not quantitatively. We thus chose the more careful approach to score only for presence/absence of transcripts of *A. thaliana/T. hassleriana* putatively orthologous gene pairs. Of the 21,107 genes in *A. thaliana* for which microarray expression data for the floral transcriptome could be compiled, ~1200 genes were not expressed in the *A. thaliana*. The expression analysis of these gene homologs in the *T. hassleriana* revealed that a majority of these genes (~750) were also not expressed in the *T. hassleriana* floral transcriptome. But 351 gene homologs were identified that were expressed differentially amongst the floral transcriptomes of the two species. These differentially expressed Tarenaya transcripts were assigned GO annotations using Blast2GO® by performing a BLASTX search with a cut off value of e^-100^ to identify the molecular processes that are distinct between *T. hassleriana* and *A. thaliana*. 81 genes were annotated as genes with unknown function. The remaining 270 genes were assigned multiple GO annotations based on the biological processes associated with the function of these genes (Additional file 1: Table S5). Of special interest were genes annotated to be involved in anthocyanin accumulation, cell growth, flower development and other developmental processes. Candidate genes were selected for further analysis (Table 2). High expression of *PGP10* homolog, a gene involved in anthocyanin accumulation in response to UV light was observed in the *T. hassleriana* floral transcriptome whereas its expression is limited to pollen in *A. thaliana*. The homolog of *TTFP* which codes for a tyrosine transaminase family protein was also expressed at high levels in the *T. hassleriana* floral transcriptome; this gene is involved in regulation of cell growth in response to external stimulus and is primarily expressed in the roots of *A. thaliana*. Other notable gene homologs involved in various aspects of cell growth were *LRX2, HAT4* and *PIP5K3*. Of the gene homologs involved in various aspects of floral development, prominent were *ICMTA* and *TEM2. ICMTA* is an enzyme belonging to the methyltransferase family, which is inducted during floral morphogenesis. *TEM2* is a transcription factor known for its role in flowering time regulation by controlling *FT* expression. Amongst the genes annotated as genes governing various aspects of development were *JAL33, MTSP1*, *EMB2217* and *GLUDOXRP* which are involved in embryo and root development.

**Table 2:** **Selection of homologous gene pairs in which the homologs of *T. hassleriana *** **are expressed in the flower and the *A. thaliana *** **homolog expression is absent from the flower**

**Gene abbreviation**	**Process/protein family**	**GO ID**	***T. hassleriana *** **TSE (RPKM)**	**Expression in *A. thaliana***
Anthocyanin accumulation				
*PGP10*	multidrug pheromone mdr abc transporter family	GO:0043481	63.79	Mature pollen
Cell growth				
*TTFP*	tyrosine transaminase family protein	GO:0001560	194.20	Root
*ARP2*	actin-related protein 2-like	GO:0009825	28.21	Senescent leaf, cauline leaf, buds, flower, inflorescence shoot apex
*HB-2*	homeodomain-leucine zipper protein	GO:0009826	28.07	Young leaf, mature leaf, cauline leaf, senescent leaf, pedicel, seed (torpedo stage embryo)
*LRX2*	leucine-rich repeat extensin-like protein 1	GO:0009826	22.97	Young leaf, pollen, seed (cotyledon stage embryo)
*PLLSP*	pectate lyase family protein	GO:0042547	12.85	Young leaf petiole, mature leaf (distal end), seed (curled cotyledon stage embryo)
Flower development				
*ICMTA*	protein-s-isoprenylcysteine o-methyltransferase a	GO:0009908	37,04	Young leaf, cauline leaf, senescent leaf, young silique, seed (heart and torpedo stage embryo)
*SBP3*	selenium-binding protein	GO:0048573	24,26	Imbibed seed
*BTB/POZ P*	BTB/POZ domain-containing protein	GO:0048439	12,55	Petals stamens
*TEM2*	ap2 erf and b3 domain-containing transcription factor rav2	GO:0009910	12,28	Cotyledon, young leaf, senescent leaf
Development				
*JAL33*	jacalin-like lectin domain-containing protein	GO:0009793	302,33	Root, hypocotyl
*MTSP2*	caffeoyl- o-methyltransferase	GO:0048316	47,03	Seed (curled cotyledon, green cotyledon embryo stage), dry seed
*MTSP1*	s-adenosyl-l-methionine-dependent methyltransferase-like protein	GO:0010089	40,59	Seed (walking stick, curled cotyledon, green cotyledon embryo stage)
*LRRTPKP*	lrr receptor-like serine threonine-protein kinase rch1-like	GO:0048443	15,26	Root, seed (torpedo stage embryo), imbibed seed
*CYP705A27*	cytochrome p450	GO:0048589	11,86	Root, seed (cotyledon embryo stage), dry seed
*EMB2271*	u3 small nucleolar rna-interacting protein 2-like	GO:0009553	11,81	Stamen
*CYP705A*	cytochrome p450	GO:0048589	11,54	Root
*GLUDOXRP*	glutaredoxin-related protein	GO:0048653	7,40	Pollen, seed (walking stick, curled cotyledon, green cotyledon stage embryo)
*LRRRPK*	receptor-like protein kinase 2-like	GO:0048443	3,50	Imbibed seed, root

### Identification and characterization of Cleome lineage specific genes

**Figure 6:**
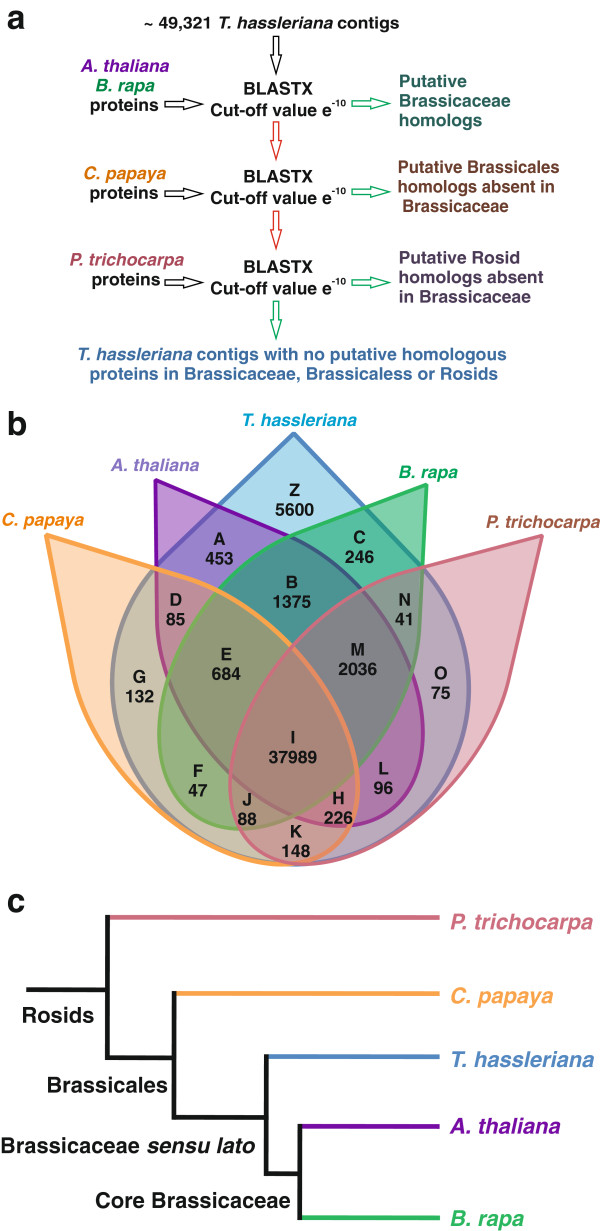
**Identification and representation of lineage specific genes in selected rosid taxa.** 6**a**: Strategy for the identification of lineage specific genes by BLASTX based sequence comparisons; 6**b**: Venn diagram representing lineage specific genes and genes shared between the different Rosid lineages, the letters in bold indicate the subset of sequences and the number indicates the number of genes/contigs included in the subset.; B: Genes shared between *A. thaliana, B. rapa* (core Brassicaceae) and *T. hassleriana*; E: Genes shared between *C. papaya, A. thaliana, B. rapa* and *T. hassleriana* (Brassicales); I: Genes shared by *A. thaliana, B. rapa, C. papaya, P. trichocarpa* and *T. hassleriana* (all Rosids); K: Genes shared by *C. papaya, P. trichocarpa* and *T. hassleriana* (potentially lost in Brassicaceae); O: Genes shared by *P. trichocarpa* and *T. hassleriana* (Lost in Brassicales); Z: contigs representing genes present only in *T. hassleriana* not shared with the other lineages. 6**c**: A schematic representation of the relationship of rosids used for the identification of Tarenaya specific genes.

To identify genes shared between Cleome and other closely related rosids and genes that are specific to the Cleome lineage a BLASTX search with a cut off value of e^-10^ was performed with the 49,237 Tarenaya floral transcriptome contigs against the *A. thaliana, Brassica rapa, C. papaya* (all malvids, order Brassicales) and *Populus trichocarpa* (fabid, order Malpighiales) protein databases in a systematic manner (Figure 6a). This allows the assessment of gene births and gene losses in the rosid lineage. Figure 6b shows the result of the comparative analysis: A large number of the contigs 37,989 (subset I) represent the sequences shared between malvids and fabids. According to our analysis, only 684 genes are shared between all Brassicales, but 1375 genes (subset B) are shared between the core Brassicales, which include *T. hassleriana, A. thaliana,* and *B. rapa*[36]. This suggests a high rate of gene births in the lineage leading to core Brassicales after their split from the lineage leading to *C. papaya*. Conversely, 148 genes (subset K) are shared between *T. hassleriana, C. papaya* and *P. trichocarpa* and not found in the Brassicaceae suggesting that these genes were lost in the lineage leading to *A. thaliana* and *B. rapa* after its separation from the lineage leading to *T. hassleriana*. Another 132 (subset G) genes are found only in *C. papaya* and *T. hassleriana* indicating that these are Brassicales-specific genes that were lost in the Brassicaceae. 453 genes are shared between *T. hassleriana* and *A. thaliana* but not found in *B. rapa* suggesting that they were lost in the lineage leading to *B. rapa*. Conversely, only 246 genes were lost in the lineage leading to *A. thaliana* and are shared between *B. rapa* and *T. hassleriana* (subset C).

An astonishing number of 5600 contigs (subset Z) could not be matched with high confidence to any other sequence from *P. trichocarpa, C. papaya, A. thaliana* and *B. rapa*. Of these contigs only 82 could be assigned to 353 GO terms, but a vast majority of the contigs could not be annotated attributing to no significant BLAST hits. A sequence length histogram for these contigs (Additional file 2: Figure S2) shows a bias towards shorter sequences when compared to the sequence length histogram of all contigs (Additional file 2: Figure S1b) suggesting that these were too short for proper annotation and/or may represent 5’ and 3’ UTR regions of transcripts. Another reason for the small number of annotated genes is because most of the current annotations are based on *A. thaliana*, *B. rapa* and *P. trichocarpa* and we already subtracted the sequences orthologous to them. The GO annotations for the *T. hassleriana* specific genes are the following: cellular process (26.98%), metabolic process (29.36%), response to stimulus (4.76%), biological regulation (4.7%), development (1.5%), cell proliferation (1.5%), reproduction (6.34%) and signaling processes (4.76%) (Additional file 1: Table S6).

## Discussion

In this work we present the floral transcriptome sequence of *T. hassleriana*, which is a member of the Cleomaceae and thus a sister taxon to the Brassicaceae. The transcriptome was analyzed by rarefaction analysis and shown to be of sufficient depth to also identify rare transcripts. As normalization was not carried out, abundance of transcripts could be assessed in silico and compared to qRT-PCR data. The leaf transcriptome of *T. hassleriana* has been published earlier [10] allowing for comparison of transcript abundance between the leaf and the floral transcriptome. We also attempted to compare the expression of genes represented in the floral transcriptome with the expression of their respective *A. thaliana* orthologs based on presence/absence of expression in the microarray dataset [22] including all flower developmental stages. In addition, we are able to identify 5600 putative transcripts that are specific to the Cleomaceae and 684, which are shared only among the Brassicales *C. papaya*, *T. hassleriana, B. rapa*, and *A. thaliana*.

During assembly, annotation and analysis of the reads obtained by 454 sequencing we observed several challenges. In our study we correlated the in silico floral transcriptome expression in *T. hassleriana* with the conventional qRT-PCR expression of arbitrarily chosen genes with low, moderate, and high expression levels for validation of the transcriptome sequencing expression data. For the in silico expression data we applied two approaches, one was to map the individual reads to the annotated A. thaliana CDS sequences (TSE1), with the advantage that expression data for putative *T. hassleriana* orthologs of *A. thaliana* genes can be generated without a prior genome sequence information of *T. hassleriana*, thus individual reads are not assembled into contigs. The second approach was the de novo assembly of the reads into contigs that are then annotated and the reads are mapped on to these contigs (TSE2). Comparing both methods with qRT-PCR data, the TSE1 approach clearly matches better than the TSE2 approach. One reason for this finding is the presence of chimeric contigs composed of more than one gene, in such cases reads to multiple genes are mapped onto the same contig flaring up the expression. This problem is avoided in TSE1 when the reads are mapped onto orthologous sequences in *A. thaliana*. Another reason for the disparity between the qRT-PCR expression and TSE2 is due to assembly of contigs with additional non coding nucleotide sequences. This phenomenon was observed in the case of *RBCS1A* amongst the genes analyzed. The assembled contig was 838 nucleotides long whereas the coding sequence of this gene is ~540 nucleotides across many plant lineages. The additional 295 nucleotides at the 3’ end could represent the 3’ UTR nonetheless reads would be mapped to such sequences leading to an overestimation of expression. The third reason for the differences in the expression between qRT-PCR and TSE2 may be the length of the assembled contig versus transcript size, as larger transcripts are fragmented prior cDNA library preparation. For TSE1, qRT-PCR expression data were normalized to *A. thaliana* CDS lengths and in case of TSE2 to *T. hassleriana* contig length. The necessity of normalization was seen in case of gene *SPL7*. The *SPL7* contig length was 629 nucleotides whereas the coding sequence of *SPL7* in *A. thaliana* is 2406 nucleotides, thus when the expression is normalized for length of the contig it leads to a much higher expression than when normalized to the length of the *A. thaliana* ortholog. In case where the contig length matched to the coding sequence length and when the contig had very low or almost no unknown sequences incorporated the qRT-PCR expression matched very well to the TSE2 as was observed in case of *TT4*.

However, since *T. hassleriana* and *A. thaliana* have, independent α-WGDs, the retention and loss of gene copies following the duplication will be different. By mapping the *T. hassleriana* reads onto the *A. thaliana* orthologs identical sets of orthologous gene copies are assumed for both species leading to over or underestimation of transcript abundance. These illustrated pitfalls for calculating gene expression from RNAseq experiments without the availability of a high-quality reference transcriptome or genome require thorough independent validation of gene expression data.

This work was initiated as a primer to identify genes that may contribute to the morphological differences between the *T. hassleriana* and the *A. thaliana* flower. Our focus was mainly on coloration, flowering time, and floral organ size as these are traits that show obvious differences between the two species.

*T. hassleriana* petals show a deep pink coloration which, when the flower opens, fades into light pink after a few days of exposure to the sun. While in most species only the epidermal petal cell layer is pigmented, *T. hassleriana* also has pigmented mesophyll cells [14]; suggesting an expansion of the anthocyanin regulation and biosynthesis pathway from petal to mesophyll cells. The pink pigments found in *T. hassleriana* flowers are acetylated cyanidin diglucoside (sophorosyl)-5-glucosides and acetylated pelargonidin sophorosyl-5-glucosides [14]. All genes required for the synthesis of pelargonidin-3-glucoside and cyanidin-3-glucoside are present in the flower transcriptome. Genes encoding proteins required for the early steps of anthocyanin up to the flavonoid myricetin are also found expressed in the leaf transcriptome, while the genes participating in later steps such as *DFR* and *LDOX* are restricted to the flower. These two enzymes are also not expressed in *A. thaliana* flowers but during seed development and late stages of leaf senescence [32].

Transcription factors of the MYB, bHLH, and WD40 families regulate the expression of anthocyanin biosynthesis genes in *A. thaliana* and Zea mays. While early biosynthesis genes, and their regulators such as *AtMYB11*, *AtMYB12*, and *AtMYB111* are involved in the production of flavonols, late biosynthesis genes and their regulators are required for the synthesis of anthocyanins from flavonols [37] and references therein. While the putative T. hassleriana orthologs of *AtMYB11* and *AtMYB12* are hardly expressed in the flower transcriptome, the putative *AtMYB111* ortholog shows very strong and flower specific expression suggesting a more prominent role for this gene in the regulation of early biosynthesis genes than for the putative orthologs of *AtMYB11* and *AtMYB12*. Orthologs of the regulators of late anthocyanin biosynthesis in *A. thaliana AtTTG1* (WD40 family member), *AtTT8, AtGL3, AtEGL3* (all bHLH family members) and *AtPAP2* (MYB family member) are also found expressed in the *T. hassleriana* flowers. The *T. hassleriana* orthologs of *A. thaliana* genes *AtTTG1, AtTT8, AtGL3, AtEGL3*, and *AtPAP2* forming the late anthocyanin biosynthesis regulatory complex show an approximately similar transcript abundance suggesting that they may function in a complex similar to the one in *A. thaliana*, only with an expression domain expanded to the floral organs.

*A. thaliana* late regulators are mainly expressed in senescing leaves and during seed development (Table 2), but most likely, their expression domain in *T. hassleriana* has expanded into the flower leading to the pink coloration of the floral organs. A similar situation is found in petunia, where, among others genes *AN1, AN11, AN2* and *AN4* form complexes similar to that in *A. thaliana* to regulate anthocyanin biosynthesis in the flower [38].

*T. hassleriana* has, unlike *A. thaliana*, large oval shaped petals, and indeed orthologs of genes involved in limiting growth of floral organs were found to be hardly expressed in the *T. hassleriana* floral transcriptome. *BIG BROTHER*, encoding for a E3 ubiquitin-ligase represses cell proliferation in all *A. thaliana* proliferating tissues and is expressed strongly and uniformly in all developmental stages of the flower independently of other pathways while being a direct target of the petal organ identity gene *AP3*[39-41]. In the *T. hassleriana* floral transcriptome it has a very low expression of 9 RPKM, suggesting that this may be a reasonable candidate to account for the differences in petal size between the two species.

The particular *T. hassleriana* hybrid used in this study is sterile even though it produces all the floral organ whorls in the right number and position. However, even though the anthers developed, they did not produce any pollen and also did not dehisce rendering the plants male sterile. While orthologs of *A. thaliana* regulators of anther development were expressed in the the *T. hassleriana* flower, no expression of *ROXY1* and *ROXY2* was detected. These two genes redundantly control the anther lobe and pollen mother cell differentiation downstream of *SPL* in *A. thaliana*[34]. Moreover, only very low expression (6 RPKM) was observed for the *T. hassleriana* ortholog of *DYT1* which acts directly downstream of the *ROXY* genes. The phenotype of the *T. hassleriana* anthers also resembles the *roxy1 roxy2* double mutant anther phenotype in *A. thaliana*, suggesting that our *T. hassleriana* hybrid may lack functional *ROXY* genes leading to male sterility. We corroborated this observation by qRT-PCR expression data which not only detected very low *ROXY1* expression (the only *ROXY* ortholog in *T. hassleriana* genome [35]) in the mid developmental stage but also showed the de-regulation of expression of the upstream and downstream genes throughout bud development which may provide a cause for the male sterility.

*T. hassleriana* is perpetually flowering and a sharp transition to flowering as in *A. thaliana* cannot be observed. Several genes involved in flowering time regulation in *A. thaliana* are differently regulated in leaves and flowers and we compared the expression of their orthologs in the flower and leaf transcriptomes. FRI is a protein involved in activating transcription via chromatin remodeling of the central floral repressor *FLC* in *A. thaliana*[42] and is expressed rather uniformly throughout the plant. However, in *T. hassleriana*, *FRI* ortholog expression is not found in leaves. This may suggest a different mechanism for *FLC* ortholog activation in *T. hassleriana* leaves, as *FLC* is expressed there without the presence of *FRI*.

Interestingly; the expression of two more genes most likely involved in the change from vegetative to reproductive phase in *T. hassleriana* is different from *A. thaliana*. The *A. thaliana* gene *SMZ* is expressed in young seedlings, during floral transition and seed maturation [43] unlike its *T. hassleriana* ortholog which is expressed in flowers and developing buds. Possibly, the *T. hassleriana SMZ* has function different from its *A. thaliana* ortholog, which is a rather strong repressor of flowering.

Another candidate gene in the group of flowering time regulators that are differentially regulated in *A. thaliana* and *T. hassleriana* is *ATC*. In *A. thaliana*, *ATC* is strongly expressed in the root and a small fraction (1-5%) of its mRNAs moves a long distance to the plant’s apex [44]. Notably, we find a significant amount of reads in flower tissue, too many to attribute them to long distance RNA transport. More likely, the *ATC* homolog is expressed in *T. hassleriana* floral tissue and may be transported throughout the plant to enable the vegetative shoots to first reach sufficient size to start flowering.

## Conclusions

Taken together we conclude from our expression data that a number of floral regulators show expression distinct from that in *A. thaliana* suggesting that differences in life history traits such as perpetual flowering and pigmentation may be regulated by similar components of regulatory networks in *A. thaliana* and *T. hassleriana* that are highly conserved in coding sequence but expressed in a different way in the two species, suggesting that modifications in expression pattern account for a large part of the diversity in flowers and plant life history traits.

## Abbreviations

WGD: Whole genome duplication; At-α: *A. thaliana* alpha WGD; At-β: *A. thaliana* beta WGD; Cq: Quantification cycle; EST: Expressed sequence tag; MYA: Million years ago; RPKM: Reads per kilobase gene model per mappable million; SDR: Standard dose response; Th-α: *T. hassleriana* alpha WGD; TSE: Transcriptome sequencing expression; TSE1: TSE mapped to *A. thaliana* CDS sequences; TSE2: TSE mapped to *T. hassleriana* contigs.

## Competing interests

The authors declare that they have no competing interests.

## Authors’ contributions

A. Bhide performed the molecular biology experiments, comparative and in silico expression analysis and drafted the manuscript. SS carried out RNAseq assembly, annotation, in silico expression analysis, lineage specific gene discovery. MR performed the rarefaction analysis, A. Becker coordinated and designed the study, and helped to draft the manuscript. A. Becker and APMW conceived the study. All authors helped to improve the manuscript, read, and approved of the final manuscript.

## Supplementary Material

Additional file 1: Table S1 454 sequencing statistics. **Table S2.** Sequences of the oligonucleotides used for the qRT-PCR. **Table S3.** List of gene names along with their abbreviations and AGI identifiers. **Table S4.** P- value calculations using one way ANOVA for analyzing the statistical significance of difference between expression values by qRT-PCR and Transcriptome Sequencing Expression (TSE). **Table S5.** GO annotation of putatively homologous gene pairs expressed in the *T. hassleriana* floral transcriptome but not expressed in the *A. thaliana* floral transcriptome. **Table S6.** GO annotation of *T. hassleriana* specific sequences not found in *A. thaliana*, *B. rapa*, *C. papaya*, and *P. trichopoda* using Blast2GO® with BLASTX searches. Click here for file

Additional file 2: Figure S1 454 sequencing statistics. **Figure S2.** Read length distribution of *T. hassleriana* lineage specific contigs without any GO annotation. **Figure S3.** Correlation plot of TSE1 expression by RNA seq and qRT-PCR gene expression. Click here for file

Additional file 3 ***T. hassleriana* floral transcriptome gene expression.** Click here for file
