# Supplementary material for: Analysis of the floral transcriptome of Tarenaya hassleriana (Cleomaceae), a member of the sister group to the Brassicaceae: towards understanding the base of morphological diversity in Brassicales
Source: BMC Genomics. 2014 Feb 19;15:140. doi: 10.1186/1471-2164-15-140 (PMC4028054; doi:10.1186/1471-2164-15-140)
Supplement: Additional file 1: Table S1 — 454 sequencing statistics. Table S2. Sequences of the oligonucleotides used for the qRT-PCR. Table S3. List of gene names along with their abbreviations and AGI identifiers. Table S4. P- value calculations using one way ANOVA for analyzing the statistical significance of difference between expression values by qRT-PCR and Transcriptome Sequencing Expression (TSE). Table S5. GO annotation of putatively homologous gene pairs expressed in the T. hassleriana floral transcriptome but not expressed in the A. thaliana floral transcriptome. Table S6. GO annotation of T. hassleriana specific sequences not found in A. thaliana, B. rapa, C. papaya, and P. trichopoda using Blast2GO® with BLASTX searches. [file 1471-2164-15-140-S1.docx]

**Additional File 1**

**Table S1:** 454 sequencing statistics depicting total number of reads for the two replicates and the contig assembly and mapping statistics. The runs were mapped to TAIR10 coding sequences (cds) for quantitative expression data. Contigs were assembled de novo using CLC Genomics Workbench

|  | sample 1 | sample 2 | total |
| --- | --- | --- | --- |
| No. of reads | 598930 | 655356 | 1254286 |
| average read length [nt] | 333,48 | 299,47 | 316,475 |
| No. mapped to TAIR10 cds | 259938 | 307966 | 567904 |
| Percentage mapped to TAIR10 cds | 43,40% | 46,99% | 45,20% |
| No. of contigs | 49237 | | |
| N50 | 690 | | |
| No. of chimeric contigs | 537 | | |

**Table S2:** Sequences of the oligonucleotides used for the qRT-PCR.

| **Primer** | **5'-3' sequence** |
| --- | --- |
| ACT7_F | TCCACGAAACCACATACAACTC |
| ACT7_R | TATCCACATCTGCTGGAAGGT |
| RBCS1A_F | GTCCGCAGTGATTGCTCCG |
| RBCS1A_R | ATGCGTTCCCCAACTCGAA |
| MVP1_F | GAAGGAAATTCGCCCTCCAG |
| MVP1_R | TCCCTTCTCAAACGTGTAAGTC |
| GAPC1_F | CCTTCAACATTATTCCTAGCAGCA |
| GAPC1_R | TTCTGACTCCTCCTTGATAGCC |
| TT4_F | GACTACTACTTCCGCATCACCAAC |
| TT4_R | CTCCTTGATGGCTCTGACCG |
| BGLUC19_F | GGATCACATTGAACGAGCCA |
| BGLUC19_R | ACCTTTGCACTTCTCACACTG |
| GAMMAVPE_F | GGTGTTCCGAAGGATTACACTG |
| GAMMAVPE_R | GCATACAGGTAAGGAGAAGTTGG |
| ATP3_F | AGCTGTCTTCTGGTCCTGAG |
| ATP3_R | GTTGGTAGGAACGCCATCAC |
| SCE1A_F | GGGAAATCTGGCACTGACTG |
| SCE1A_R | CGAGTAAATCCTGAATACCAACCA |
| SFGH_F | AAGAGTTGCCAAAGCTCCTG |
| SFGH_R | CATCATATTCCTCCCAATCAGCC |
| ARF6_F | GTTTCCTCCACTGGACTATACAC |
| ARF6_R | GCCTTATACCGAGAAGTAGCTG |
| PGLUHYD_F | CTCGGAATCTATTCAGGTGGAC |
| PGLUHYD_R | AATGACTCAATTCCAGTGCCA |
| GI_F | ACTAGCAACTCTGGGAAGGG |
| GI_R | GCATCAACTACTTGTCTCCATCC |
| OMR1_F | CGGAAATCAAGTGGCAATCTG |
| OMR1_R | CAAAGGACCCTTAGCTTGGC |
| SPL7_F | GGCATCAAATATTCCAATGGCTG |
| SPL7_R | GTTCAAATAAACAGTCATCGAGCC |
| MYB35_F | ATTCTTGCTTATGTCTCCATCCAC |
| MYB35_R | CAATCAAAGACCACCTGCTACC |
| SPL_F | GTGCCGAAGATCAACAGGAG |
| SPL-R | GTCGTAACGTTTCTTCTTGAAGC |
| ROXY1_F | AGAGCAGTAGTGATATTCAGCG |
| ROXY1_R | ATAGCTCCAAGGAGTTTGCC |
| DYT1_F | TCACCAACATGAAGAAAGCGA |
| DYT1_R | TCTTACTCACATGAACATCTTCCC |

**Table S3:** List of gene names along with their abbreviations and AGI identifiers

| AGI Indentifier | *Gene name* | *abbreviation* |
| --- | --- | --- |
| AT5G09810 | *ACTIN 7* | *ACT7* |
| AT2G45190 | *ABNORMAL FLORAL ORGANS* | *AFO* |
| AT4G18960 | *AGAMOUS* | *AG* |
| AT4G24540 | *AGAMOUS-LIKE 24* | *AGL24* |
| AT2G45660 | *AGAMOUS-LIKE 20* | *AGL20* |
| AT4G37750 | *AINTEGUMENTA* | *ANT* |
| AT1G69120 | *APETALA1* | *AP1* |
| AT4G36920 | *APETALA 2* | *AP2* |
| AT3G54340 | *APETALA 3* | *AP3* |
| AT1G30330 | *AUXIN RESPONSE FACTOR 6* | *ARF6* |
| AT3G59900 | *AUXIN-REGULATED GENE INVOLVED IN ORGAN SIZE* | *ARGOS* |
| AT3G27000 | *ACTIN RELATED PROTEIN 2* | *ARP2* |
| AT5G42190 | *ARABIDOPSIS SKP-LIKE 2* | *ASK2* |
| AT2G27550 | *CENTRORADIALIS* | *ATC* |
| AT2G33040 | *GAMMA SUBUNIT OF MT ATP SYNTHASE* | *ATP3* |
| AT2G31650 | *HOMOLOG OF ANTI-OXIDANT 1* | *ATX1* |
| AT5G65700 | *BARELY ANY MERISTEM 1* | *BAM1* |
| AT3G49670 | *BARELY ANY MERISTEM 2* | *BAM2* |
| AT4G20270 | *BARELY ANY MERISTEM 3* | *BAM3* |
| AT3G63530 | *BIG BROTHER* | *BB* |
| AT3G21370 | *BETA GLUCOSIDASE 19* | *BGLUC19* |
| AT2G41370 | *BLADE ON PETIOLE2* | *BOP2* |
| AT3G57130 | *BLADE ON PETIOLE 1* | *BOP1* |
| AT2G01930 | *BASIC PENTACYSTEINE1* | *BPC1* |
| AT1G59640 | *BIG PETAL P* | *BPEP* |
| AT2G46020 | *BRAHMA* | *BRM* |
| AT1G04390 | *BTB/POZ DOMAIN CONTAINING PROTEIN* | *BTB/POZ P* |
| AT1G26310 | *CAULIFLOWER* | *CAL* |
| AT5G62430 | *CYCLING DOF FACTOR 1* | *CDF1* |
| AT5G05170 | *CONSTITUTIVE EXPRESSION OF VSP 1* | *CEV1* |
| AT5G05270 | *CHALCONE ISOMERASE LIKE* | *CHIL* |
| AT2G23380 | *CURLY LEAF* | *CLF* |
| AT1G75820 | *CLAVATA 1* | *CLV1* |
| AT1G65380 | *CLAVATA 2* | *CLV2* |
| AT5G15850 | *CONSTANS-LIKE 1* | *COL1* |
| AT3G02380 | *CONSTANS-LIKE 2* | *COL2* |
| AT3G07650 | *CONSTANS-LIKE 9* | *COL9* |
| AT1G69180 | *CRABS CLAW* | *CRC* |
| AT1G04400 | *CYRPTOCHROME 2* | *CRY2* |
| AT1G76420 | *CUP SHAPED COTYLEDON3* | *CUC3* |
| AT5G53950 | *CUP-SHAPED COTYLEDON 2* | *CUC2* |
| AT5G04630 | *CYTOCHROME P450, FAMILY 77, SUBFAMILY A, POLYPEPTIDE 9* | *CYP77A9* |
| AT1G50520 | *CYTOCHROME P450, FAMILY 705, SUBFAMILY A, POLYPEPTIDE 27* | *CYP705A27* |
| AT1G50560 | *CYTOCHROME P450, FAMILY 705, SUBFAMILY A, POLYPEPTIDE 25* | *CYP705A* |
| AT5G42800 | *DIHYDROFLAVONOL 4-REDUCTASE* | *DFR* |
| AT4G21330 | *DYSFUNCTIONAL TAPETUM 1* | *DYT1* |
| AT4G22140 | *EARLY BOLTING IN SHORT DAYS* | *EBS* |
| AT1G63650 | *ENHANCER OF GLABRA 3* | *EGL3* |
| AT4G21130 | *EMBRYO DEFECTIVE 2271* | *EMB2271* |
| AT5G51230 | *EMBRYONIC FLOWER 2* | *EMF2* |
| AT4G31820 | *ENHANCER OF PINOID* | *ENP* |
| AT2G26330 | *ERECTA* | *ER* |
| AT5G40280 | *ENHANCED RESPONSE TO ABA 1* | *ERA1* |
| AT5G62230 | *ERECTA-LIKE 1* | *ERL1* |
| AT5G07180 | *ERECTA-LIKE 2* | *ERL2* |
| AT2G33860 | *ETTIN* | *ETT* |
| AT1G53520 | *FATTY-ACID-BINDING PROTEIN 3* | *FAP3* |
| AT3G51240 | *FLAVANONE 3-HYDROXYLASE* | *F3H* |
| AT5G10140 | *FLOWERING LOCUS C* | *FLC* |
| AT3G10390 | *FLOWERING LOCUS D* | *FLD* |
| AT3G04610 | *FLOWERING LOCUS KH DOMAIN* | *FLK* |
| AT5G08640 | *FLAVONOL SYNTHASE 1* | *FLS1* |
| AT4G00650 | *FRIGIDA* | *FRI* |
| AT1G65480 | *FLOWERING LOCUS T* | *FT* |
| AT3G59380 | *FARNESYLTRANSFERASE A* | *FTA* |
| AT1G14920 | *GIBBERELLIC ACID INSENSITIVE* | *GAI* |
| AT4G32940 | *GAMMA VACUOLAR PROCESSING ENZYME* | *GAMMAVPE* |
| AT3G04120 | *GLYCERALDEHYDE-3-PHOSPHATE DEHYDROGENASE C SUBUNIT 1* | *GAPC1* |
| AT1G22770 | *GIGANTEA* | *GI* |
| AT5G41315 | *GLABROUS 3* | *GL3* |
| AT3G11920 | *GLUTAREDOXIN RELATED PROTEIN* | *GLUDOXRP* |
| AT4G16780 | *HOMEOBOX PROTEIN 2* | *HB-2* |
| AT3G12680 | *ENHANCER OF AG-4 1* | *HUA1* |
| AT5G23150 | *ENHANCER OF AG-4 2* | *HUA2* |
| AT5G23320 | *ISOPRENYL CYSTEINE METHYLTRANSFERASE A* | *ICMTA* |
| AT5G67100 | *INCURVATA2* | *ICU2* |
| AT1G23420 | *INNER NO OUTER* | *INO* |
| AT1G68480 | *JAGGED* | *JAG* |
| AT3G16450 | *JACALIN-RELATED LECTIN 33* | *JAL33* |
| AT1G70510 | *KNOTTED-LIKE FROM ARABIDOPSIS THALIANA 2* | *KNAT2* |
| AT4G22880 | *LEUCOANTHOCYANIDIN DIOXYGENASE* | *LDOX* |
| AT5G61850 | *LEAFY* | *LFY* |
| AT3G24240 | *LEUCINE-RICH REPEAT RECEPTOR-LIKE PROTEIN KINASE FAMILY PROTEIN* | *LRRRPK* |
| AT5G48940 | *LEUCINE-RICH REPEAT TRANSMEMBRANE PROTEIN KINASE FAMILY PROTEIN* | *LRRTPKP* |
| AT1G62440 | *LEUCINE-RICH REPEAT/EXTENSIN 2* | *LRX2* |
| AT4G32551 | *LEUNIG* | *LUG* |
| AT2G32700 | *LEUNIG_HOMOLOG* | *LUH* |
| AT5G04380 | *METHYL TRANSFERASE SUPERFAMILY PROTEIN 1* | *MTSP1* |
| AT1G24735 | *METHYL TRANSFERASE SUPERFAMILY PROTEIN 2* | *MTSP2* |
| AT1G54030 | *MODIFIED VACUOLE PHENOTYPE 1* | *MVP1* |
| AT3G28470 | *MYB DOMAIN PROTEIN 35* | *MYB35* |
| AT5G49330 | *MYB DOMAIN PROTEIN 111* | *MYB111* |
| AT1G66370 | *MYB DOMAIN PROTEIN 113* | *MYB113* |
| AT3G62610 | *MYB DOMAIN PROTEIN 11* | *MYB11* |
| AT2G47460 | *MYB DOMAIN PROTEIN 12* | *MYB12* |
| AT5G49330 | *MYB DOMAIN PROTEIN 111* | *MYB111* |
| AT4G28530 | *NAC DOMAIN CONTAINING PROTEIN 74* | *NAC074* |
| AT1G13400 | *NUBBIN* | *NUB* |
| AT3G10050 | *L-O-METHYLTHREONINE RESISTANT 1* | *OMR1* |
| AT1G68640 | *PERIANTHIA* | *PAN* |
| AT1G66390 | *PRODUCTION OF ANTHOCYANIN PIGMENT 2* | *PAP2* |
| AT4G26000 | *PEPPER* | *PEP* |
| AT5G12950 | *PUTATIVE GLUCOSYL HYDROLASE* | *PGLUHYD* |
| AT1G10680 | *P-GLYCOPROTEIN 10* | *PGP10* |
| AT5G20240 | *PISTILLATA* | *PI* |
| AT2G34650 | *PINOID* | *PID* |
| AT1G73590 | *PIN-FORMED 1* | *PIN1* |
| AT2G26420 | *1-PHOSPHATIDYLINOSITOL-4-PHOSPHATE 5-KINASE 3* | *PIP5K3* |
| AT5G04310 | *PECTIN LYASE LIKE SUPERFAMILY PROTEIN* | *PLLSP* |
| AT3G02150 | *PLASTID TRANSCRIPTION FACTOR 1* | *PTF1* |
| AT5G03680 | *PETAL LOSS* | *PTL* |
| AT1G67090 | *RIBULOSE BISPHOSPHATE CARBOXYLASE SMALL CHAIN 1A* | *RBCS1A* |
| AT5G20570 | *RING-BOX 1* | *RBX1* |
| AT3G42830 | *RING/U-box superfamily protein* | *RBX1LP* |
| AT3G02000 | *ROXY1* | *ROXY1* |
| AT5G14070 | *ROXY2* | *ROXY2* |
| AT5G02030 | *REPLUMLESS* | *RPL* |
| AT5G35770 | *STERILE APETALA* | *SAP* |
| AT3G23800 | *SELENIUM-BINDING PROTEIN 3* | *SBP3* |
| AT3G57870 | *SUMO CONJUGATING ENZYME 1A* | *SCE1A* |
| AT5G15800 | *SEPALLATA1* | *SEP1* |
| AT3G02310 | *SEPALLATA2* | *SEP2* |
| AT1G24260 | *SEPALLATA3* | *SEP3* |
| AT2G03710 | *SEPALLATA4* | *SEP4* |
| AT1G43850 | *SEUSS* | *SEU* |
| AT2G41530 | *S-FORMYLGLUTATHIONE HYDROLASE* | *SFGH* |
| AT3G58780 | *SHATTERPROOF 1* | *SHP1* |
| AT2G42830 | *SHATTERPROOF 2* | *SHP2* |
| AT1G75950 | *ARABIDOPSIS SKP1 HOMOLOGUE 1* | *SKP1* |
| AT3G54990 | *SCHLAFMUTZE* | *SMZ* |
| AT2G39250 | *SCHNARCHZAPFEN* | *SNZ* |
| AT4G27330 | *SPOROCYTELESS* | *SPL* |
| AT5G18830 | *SQUAMOSA PROMOTER BINDING PROTEIN-LIKE 7* | *SPL7* |
| AT4G36930 | *SPATULA* | *SPT* |
| AT4G09960 | *SEEDSTICK* | *STK* |
| AT1G62360 | *SHOOT MERISTEMLESS* | *STM* |
| AT1G30970 | *SUPPRESSOR OF FRIGIDA4* | *SUF4* |
| AT3G23130 | *SUPERMAN* | *SUP* |
| AT3G04740 | *STRUWWELPETER* | *SWP* |
| AT3G15030 | *TCP FAMILY TRANSCRIPTION FACTOR 4* | *TCP4* |
| AT1G53230 | *TEOSINTE BRANCHED 1, CYCLOIDEA AND PCF TRANSCRIPTION FACTOR 3* | *TCP3* |
| AT1G67260 | *TEOSINTE BRANCHED 1, CYCLOIDEA AND PCF TRANSCRIPTION FACTOR 1* | *TCP1* |
| AT1G69690 | *TEOSINTE BRANCHED1/CYCLOIDEA/PCF 15* | *TCP15* |
| AT2G31070 | *TCP DOMAIN PROTEIN 10* | *TCP10* |
| AT3G18550 | *BRANCHED 1* | *TCP18* |
| AT3G27010 | *TEOSINTE BRANCHED 1, CYCLOIDEA AND PCF DOMAIN FAMILY PROTEIN 20* | *TCP20* |
| AT3G47620 | *TEOSINTE BRANCHED 1, CYCLOIDEA AND PCF 14* | *TCP14* |
| AT4G18390 | *TEOSINTE BRANCHED 1, CYCLOIDEA AND PCF TRANSCRIPTION FACTOR 2* | *TCP2* |
| AT1G68840 | *TEMPRANILLO 2* | *TEM2* |
| AT5G13930 | *TRANSPARENT TESTA 4* | *TT4* |
| AT3G55120 | *TRANSPARENT TESTA 5* | *TT5* |
| AT4G09820 | *TRANSPARENT TESTA 8* | *TT8* |
| AT5G35550 | *TRANSPARENT TESTA 2* | *TT2* |
| AT4G28410 | *TYROSINE TRANSAMINASE FAMILY PROTEIN* | *TTFP* |
| AT5G24520 | *TRANSPARENT TESTA GLABRA 1* | *TTG1* |
| AT1G30950 | *UNUSUAL FLORAL ORGANS* | *UFO* |
| AT4G14090 | *UDP-GLUCOSYL TRANSFERASE SUPERFAMILY PROTEIN* | *UGT75C1* |
| AT5G17050 | *UDP-GLUCOSYL TRANSFERASE 78D2* | *UGT78D2* |
| AT4G28190 | *ULTRAPETALA1* | *ULT1* |
| AT2G20825 | *ULTRAPETALA 2* | *ULT2* |
| AT4G16845 | *REDUCED VERNALIZATION RESPONSE 2* | *VRN2* |
| AT2G17950 | *WUSCHEL* | *WUS* |
| AT1G08465 | *YABBY2* | *YAB2* |
| AT2G26580 | *YABBY5* | *YAB5* |
| Petunia gene | *ANTHOCYANIN 1* | *AN1* |
| Petunia gene | *ANTHOCYANIN 2* | *AN2* |
| Petunia gene | *ANTHOCYANIN 4* | *AN4* |
| Petunia gene | *ANTHOCYANIN 11* | *AN11* |

**Table S4:** P- value calculations using one way ANNOVA for analyzing the statistical significance of difference between expression values by qRT-PCR and Transcriptome sequencing expression (TSE)

|  | **P-values** | |
| --- | --- | --- |
| **Genes** | TSE1 vs qRT-PCR | TSE2 vs qRP-PCR |
| *ARF6* | 0,0341 | 0,0001 |
| *PGLUHYD* | 0,5968 | 0,0079 |
| *GI* | 0,0032 | 0,0001 |
| *OMR1* | 0,0819 | 0,0000 |
| *SPL7* | 0,4437 | 0,2982 |
| *GAMMAVPE* | 0,0006 | 0,0207 |
| *ATP3* | 0,8447 | 0,0046 |
| *SCE1A* | 0,0352 | 0,0032 |
| *SFGH* | 0,0251 | 0,0001 |
| *RBCS1A* | 0,0911 | 0,0013 |
| *MVP1* | 0,0004 | 0,1037 |
| *GAPC1* | 0,0033 | 0,0037 |
| *TT4* | 0,0095 | 0,1181 |
| *BGLU19* | 0,0006 | 0,0002 |

**Table S5:** GO annotation of putatively homologous gene pairs expressed in the *T. hassleriana* floral transcriptome but not expressed in the *A. thaliana* floral transcriptome

| **GO annotation biological process level 2** | **No. of sequences**  **(BLASTX e^-100^)** |
| --- | --- |
| Biological adhesion | 1 |
| Biological regulation | 106 |
| Cell proliferation | 3 |
| Cellular component organization or biogenesis | 52 |
| Cellular process | 188 |
| Death | 12 |
| Developmental process | 67 |
| Growth | 27 |
| Immune system process | 23 |
| Localization | 67 |
| Metabolic process | 190 |
| Multi-organism process | 49 |
| Multicellular organismal process | 64 |
| Pigmentation | 4 |
| Reproduction | 38 |
| Response to stimulus | 132 |
| Rhytmic process | 3 |
| Signaling | 57 |

**Table S6:** GO annotation of *T. hassleriana* specific sequences not found in *A. thaliana*, *B. rapa*, *C. papaya* and *P. trichopoda* using Blast2GO^®^ with BLASTX searches

| **GO annotation biological process level 2** | **No. of sequences (BLASTX e^-10^)** |
| --- | --- |
| Biological regulation | 6 |
| Cell proliferation | 2 |
| Cellular component organization or biogenesis | 2 |
| Cellular process | 34 |
| Death | 1 |
| Developmental process | 2 |
| Growth | 1 |
| Localization | 8 |
| Locomotion | 2 |
| Metabolic process | 37 |
| Multi-organism process | 4 |
| Multicellular organismal process | 3 |
| Reproduction | 8 |
| Response to stimulus | 6 |
| Signaling | 4 |
| Viral reproduction | 6 |
