# Supplementary material for: Analysis of the floral transcriptome of Tarenaya hassleriana (Cleomaceae), a member of the sister group to the Brassicaceae: towards understanding the base of morphological diversity in Brassicales
Source: BMC Genomics. 2014 Feb 19;15:140. doi: 10.1186/1471-2164-15-140 (PMC4028054; doi:10.1186/1471-2164-15-140)
Supplement: Additional file 2: Figure S1 — 454 sequencing statistics. Figure S2. Read length distribution of T. hassleriana lineage specific contigs without any GO annotation. Figure S3. Correlation plot of TSE1 expression by RNA seq and qRT-PCR gene expression. [file 1471-2164-15-140-S2.pdf]

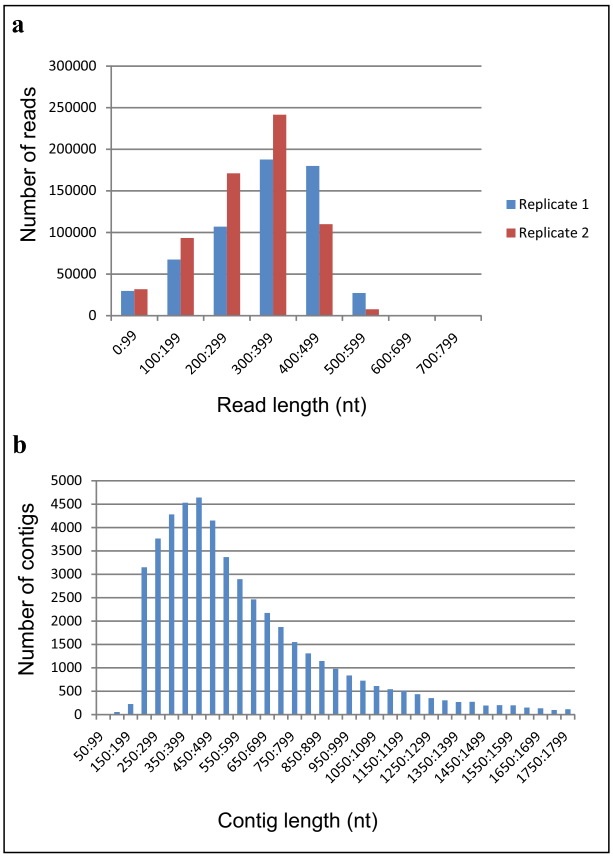

Figure S1: **454 sequencing statistics**. a: Read length distribution of individual sequencing reads, the length of the read on the X axis is plotted against the number of reads on the Y axis, replicate 1 is represented by the red bars and the replicate 2 by the blue bars. b: Read length distribution of the de novo assembled contigs; the contig length on the X axis is plotted against the number of contigs on the Y axis.

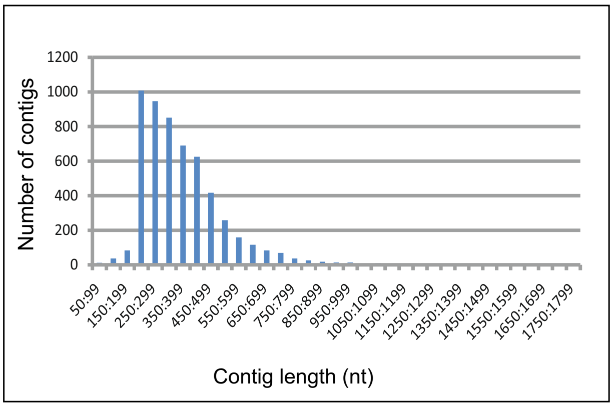

Figure S2: Read length distribution of *T. hassleriana* lineage specific contigs without any GO annotation.

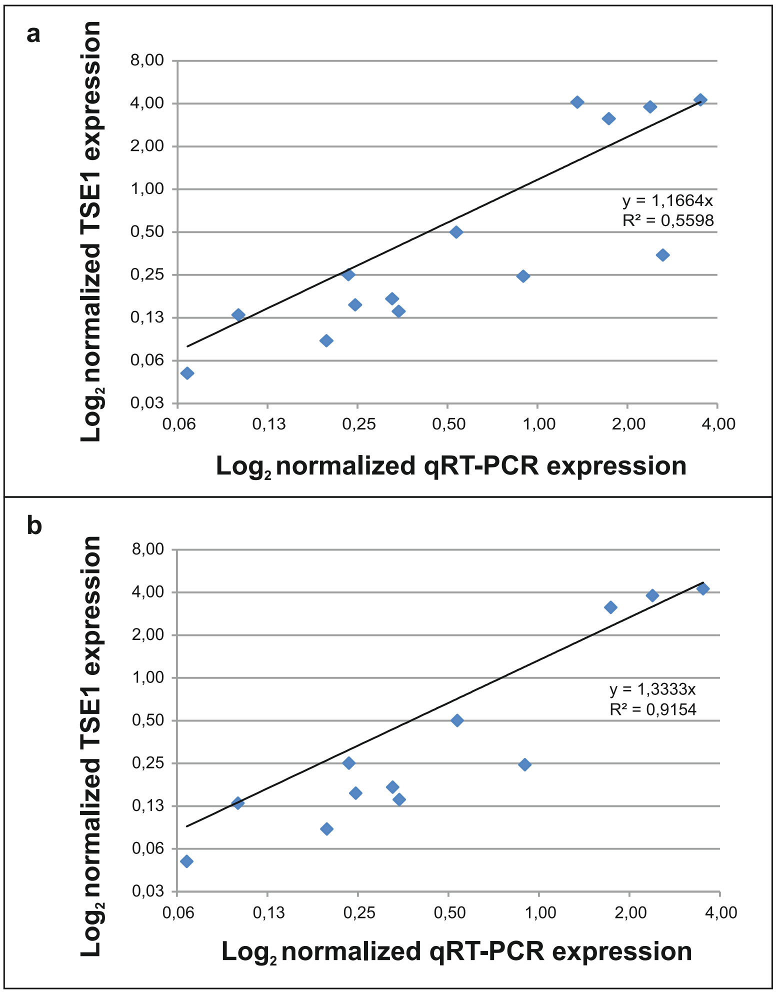

Figure S3: Correlation plot of TSE1 expression by RNA seq and qRT-PCR gene expression. a: Correlation plot for all 14 genes analysed for verification of the TSE1 expression. b: Correlation plot for 12 genes after removal of *MVP1* and *BGLUC19*.
